# Blood-Based Biomarkers: A Forgotten Friend of Hyperacute Ischemic Stroke

**DOI:** 10.3389/fneur.2021.634717

**Published:** 2021-06-08

**Authors:** Zhilan Liu, Cui Yang, Xiaoming Wang, Yang Xiang

**Affiliations:** ^1^Sichuan Provincial Center for Mental Health, Sichuan Academy of Medical Sciences & Sichuan Provincial People's Hospital, Chengdu, China; ^2^Key Laboratory of Psychosomatic Medicine, Chinese Academy of Medical Sciences, Chengdu, China; ^3^Department of Neurology, General Hospital of Western Theater Command, Chengdu, China; ^4^North Sichuan Medical College, Nanchong, China; ^5^Institute of Neurology, Sichuan Provincial People's Hospital, University of Electronic Science and Technology of China, Chengdu, China; ^6^Chinese Academy of Sciences Sichuan Translational Medicine Research Hospital, Chengdu, China; ^7^Department of Neurology, Affiliated Hospital of North Sichuan Medical College, Nanchong, China; ^8^Department of Neurology, Sichuan Provincial People's Hospital, University of Electronic Science and Technology of China, Chengdu, China

**Keywords:** hyperacute ischemic stroke, blood-based biomarker, genetic and genomic, glial activation, neuronal injury, oxidative stress, inflammation, angiogenesis

## Abstract

Ischemic stroke (IS) is the second leading cause of death worldwide. Multimodal neuroimaging techniques that have significantly facilitated the diagnosis of hyperacute IS are not widely used in underdeveloped areas and community hospitals owing to drawbacks such as high cost and lack of trained operators. Moreover, these methods do not have sufficient resolution to detect changes in the brain at the cellular and molecular levels after IS onset. In contrast, blood-based biomarkers can reflect molecular and biochemical alterations in both normal and pathophysiologic processes including angiogenesis, metabolism, inflammation, oxidative stress, coagulation, thrombosis, glial activation, and neuronal and vascular injury, and can thus provide information complementary to findings from routine examinations and neuroimaging that is useful for diagnosis. In this review, we summarize the current state of knowledge on blood-based biomarkers of hyperacute IS including those associated with neuronal injury, glial activation, inflammation and oxidative stress, vascular injury and angiogenesis, coagulation and thrombosis, and metabolism as well as genetic and genomic biomarkers. Meanwhile, the blood sampling time of the biomarkers which are cited and summarized in the review is within 6 h after the onset of IS. Additionally, we also discuss the diagnostic and prognostic value of blood-based biomarkers in stroke patients, and future directions for their clinical application and development.

## Introduction

As a disease with high incidence, disability, mortality, and recurrence rates, stroke is a significant economic burden globally because of costs associated with treatment and post-stroke care. Stroke is now the second leading cause of death worldwide (1), with Asia accounting for nearly two-thirds of the total mortality due to stroke. In China, the age-standardized prevalence, incidence, and mortality rates of stroke from 2012 to 2013 were 1.115, 0.247, and 0.115%, respectively, with ≈2.4 million new stroke cases, 1.1 million stroke-related deaths, and 11.1 million stroke survivors annually (2).

The two main subtypes of stroke are ischemic stroke (IS) and hemorrhagic stroke (HS). Management strategies for hyperacute IS include intravenous thrombolysis and intravascular treatment, for which the therapeutic time windows are <4.5 and <6 h, respectively, after symptom onset (3). Early detection and intervention are critical to ensure a good clinical outcome in hyperacute IS. A number of studies have focused on how to shorten the door-to-needle time (DNT) and door-to-puncture time (DPT). However, because laboratory and imaging examinations cannot be performed outside of the hospital setting, IS diagnosis and treatment are not possible during the pre-hospital stage, i.e., from symptom onset to on-site first aid and until the patient reaches the emergency room.

Additionally, the time nodes division standard of hyperacute IS has not been unified so far. In clinical research, the mainstream time nodes mainly include 6 or 8 h after symptom onset (4, 5). However, at present, almost all the major early diagnosis and treatment guidelines of IS recommend that intravenous thrombolysis should be carried out within 4.5 h after symptom onset, while mechanical thrombectomy should be carried out within 6 h after symptom onset. Although mechanical thrombectomy within 6–24 h after symptom onset can be carried out based on the strict inclusion criteria including endovascular therapy following imaging evaluation for ischemic stroke 3 (DEFUSE) and diffusion-weighted imaging or computed tomography perfusion assessment with clinical mismatch in the triage of wake-up and late presenting strokes undergoing neurointervention with trevo (DAWN), both of which are complex and need special imaging technique such as the rapid processing of perfusion and diffusion (RAPID) software, which significantly limits the use of mechanical thrombectomy beyond 6 h after symptom onset in the vast underdeveloped areas and community hospitals of China (6, 7).

Moreover, neuroimaging is the main technique for the diagnosis of IS in the hyperacute phase. Non-contrast computed tomography (NCCT) is the most common method used to detect stroke in the emergency room because of its simplicity, reasonable cost, and the rapidity with which results can be obtained. However, NCCT has low specificity and sensitivity for hyperacute IS (8). The development of multimodal neuroimaging techniques such as T1-, T2-, and diffusion-weighted or fluid-attenuated inversion recovery magnetic resonance imaging (MRI) has significantly facilitated diagnosis, but they are not widely used in underdeveloped areas and community hospitals in China because of their inherent shortcomings such as high cost and lack of trained operators. Additionally, current multimodal neuroimaging techniques do not have sufficient resolution to detect changes in the brain at the cellular and molecular levels after IS onset, including in cell structure, the neurotransmitter level, oxidative stress, neuroinflammation, angiogenesis, and metabolism, although these can provide important information on the prognosis of IS.

We hope that blood-based biomarkers can attract the attention of clinicians and researchers in the diagnosis of IS in the hyperacute phase, better guide clinical diagnosis, treatment, and prognosis evaluation, and supplement the shortcomings of current multimodal imaging techniques by the publication of this review manuscript. Therefore, in this review, we summarize the current state of knowledge on blood-based biomarkers of hyperacute IS including those associated with glial activation, neuronal injury, inflammation and oxidative stress, vascular injury and angiogenesis, coagulation and thrombosis, and metabolism as well as genetic and genomic biomarkers. Meanwhile, the blood sampling time of the biomarkers which are cited and summarized in the review is within 6 h after the onset of IS. Additionally, we also discuss the diagnostic and prognostic value of these biomarkers in stroke patients, and future directions for their clinical application and development.

## Biomarkers Associated with Neuronal Injury

### Ubiquitin C-Terminal Hydrolase L1 (UCH-L1)

UCH-L1, a neuronal protein that is associated with neurodegeneration, is highly expressed in the central nervous system (CNS) and is associated with synaptic plasticity, synaptic homeostasis, and self-repair of the brain after injury (9–11). Serum UCH-L1 level was elevated after 3 and 6 h of ischemia following middle cerebral artery occlusion (MCAO) in rats, while the level in intracerebral hemorrhage (ICH) rats was unchanged (12). Consistent with this finding, in rats subjected to MCAO for 30 min or 2 h, serum UCH-L1 level was significantly elevated 6 h later (13). However, a clinical study reported that serum UCH-L1 within 4 and 6 h after symptom onset was higher in HS patients than in IS patients and stroke mimics (SM), respectively (14); and in a single-center study of hyperacute IS, the area under the receiver operating characteristic curves (AUCs) of serum UCH-L1 for distinguishing IS patients from controls and IS from HS patients within 4.5 h after symptom onset were 0.64 and 0.62, respectively (15).

To sum up, the different expression of UCL-L1 in IS and HS suggests that it may be a potential biomarker in the diagnosis of hyperacute IS.

### N-Methyl-d-Aspartate Receptor (NMDAR)

Both embolic and thrombotic vascular occlusions induce neurotoxicity mediated by the excitatory NMDAR, leading to biochemical changes in brain tissue, the blood–brain barrier (BBB), and cerebral vasculature (16). Within 3 h after symptom onset, patients with IS showed elevated serum NMDAR autoantibody levels compared to healthy control subjects (HCs), which had an AUC of 0.99 for diagnosing IS with a sensitivity of 97.0% and specificity of 98.0% using the best cutoff point of 2.0 μg/l (16).

In a word, NMDAR is an independent and sensitive serologic marker capable of detecting IS with a high post-test probability, and may potentially be useful in assisting the diagnosis of hyperacute IS in the emergency setting.

### Neurofilament Light Chain (NfL)

When brain damage occurs, neuronal injury and disruption of axonal membranes lead to the release of cytoskeleton proteins, such as neurofilaments, into the interstitial fluid and eventually into the cerebrospinal fluid (CSF) and blood (17). Neurofilaments are highly specific structural, neuronal cytoskeletal proteins that consist of four neurofilament subunits: neurofilament light chain (NfL), neurofilament medium chain (NfM), neurofilament heavy chain (NfH), and α-internexin (17). In a cohort study, within 3 h after symptom onset, patients with IS showed elevated serum NfL levels compared to transient ischemic attack (TIA) (18). In summary, NfL was associated with clinical severity on admission and hyperacute IS diagnosis.

## Biomarkers Associated with Glial Activation

### Glial Fibrillary Acidic Protein (GFAP)

As an intermediate filament protein that is almost exclusively expressed in the CNS, GFAP maintains the structure and facilitates the migration of astrocytes (19). The time window between 2 and 6 h after symptom onset is critical for differentiating HS from IS based on serum GFAP level, which has a diagnostic accuracy of >80.0% (20). The AUC of plasma GFAP for differentiating HS from IS and SM within 4.5 h after symptom onset was found to be 0.915, with a sensitivity of 84.2% and specificity of 96.3% using a cutoff value of 0.29 μg/l (21); another study reported an AUC of 0.86 with a sensitivity of 61.0% and specificity of 96.0% using a cutoff of 0.34 μg/l (15). Additionally, within 4 and 6 h after symptom onset, serum GFAP had an AUC of 0.866 for differentiating HS from IS and SM, with a sensitivity of 75.0% and specificity of 84.0% using an optimal cutoff point of 72 ng/l (14); another study reported an AUC of 0.872 with a sensitivity of 77.8% and specificity of 94.2% using a cutoff of 0.03 μg/l within 6 h after symptom onset (22). A recent clinical study reported that plasma GFAP within 4.5 h after symptom onset was higher in HS patients than in IS patients (23).

In conclusion, different expression of GFAP in IS, HS, and SM suggests it may be a potential biomarker in the diagnosis of hyperacute IS; and GFAP levels were also associated with stroke severity.

### S100 Calcium-Binding Protein B (S100β)

S100β is an acidic calcium-binding protein that is predominantly expressed in astrocytes in the mammalian brain and is involved in cell cycle progression and differentiation, astrocyte–neuron communication, and CNS development and maintenance (24, 25). In a mouse model of focal cerebral infarction, plasma S100β level increased after 4 h of ischemia, which was correlated with infarction volume and the degree of neurologic deficit (26). A clinical study found that a plasma S100β concentration of 67 ng/l within the first 6 h after symptom onset could differentiate HS from IS with a sensitivity of 95.7% and a specificity of 70.4% (27). Additionally, a high serum concentration of S100β at 6 h after stroke onset was associated with increased risk of post-stroke infections (28). Another study reported that a plasma S100β concentration was lower than 1.364 pg/ml within 6 h after symptom onset could predict the development of epilepsy after a hyperacute stroke event (include IS and HS) (29).

As mentioned above, S100β may be a potential biomarker in the diagnosis of hyperacute IS; and a high serum concentration of S100β was related to severity and poor functional outcome.

## Biomarkers Associated with Inflammation and Oxidative Stress

### Cytokines

Interleukin 10 (IL-10) is an anti-inflammatory cytokine that is secreted mainly by lymphocytes and monocytes/macrophages (30). The IL-4 receptor (IL-4R) is expressed by hematopoietic, endothelial, epithelial, and muscle cells; in fibroblasts and hepatocytes; and in brain tissue, consistent with the broad target range of IL-4 (31, 32); and IL-4R binds to IL-4 to exert anti-inflammatory properties (33). Clinical-diffusion mismatch (CDM) and perfusion-diffusion mismatch (PDM) have been proposed as surrogates for the ischemic brain that is at risk of infarction. A clinical study found that along with CDM and PDM, the serum level of IL-10 at admission could identify IS patients who were candidates for thrombolytic therapy with the tissue plasminogen activator (tPA) (34). Moreover, a serum IL-10 level ≥30 pg/ml predicted a favorable functional outcome at 3 months with a sensitivity of 86.0% and specificity of 88.0% (34). Another study showed that plasma IL-4R level within 4.5 h after stroke onset was an independent predictor of poor neurologic prognosis with a sensitivity of 53.0% and specificity of 72.0% at 24 h and a sensitivity of 52.0% and specificity of 73.0% at 48 h after stroke onset using the same cutoff point of 503.40 ng/l (33).

In short, cytokines (including IL-10 and IL-4R) are independent predictors of poor neurologic prognosis and functional outcome.

### Neutrophils, Neutrophil to Lymphocyte Ratio (NLR), and Platelet to Lymphocyte Ratio (PLR)

NLR and PLR have recently been reported as potential novel biomarkers of the baseline inflammatory process and could serve as outstanding predictors in patients with IS (35, 36). Before intravenous thrombolysis treatment, the AUCs of NLR and PLR for predicting post-thrombolysis early neurological deterioration and NLR for predicting post-thrombolysis early neurological improvement were 0.763, 0.703, and 0.695, respectively (37). Additionally, within 4.5 h after symptom onset, serum neutrophil counts and NLR were positively correlated with IS severity, and higher neutrophil counts and NLR were independently associated with worse outcomes and higher mortality rates at month 3 (38).

Anyway, neutrophils, NLR, and PLR may be potential biomarkers in assessing the severity and prognosis of hyperacute IS.

### Serum Amyloid Protein (SAP)

SAP is a member of the pentraxin family and plays a key role in innate immunity and inflammation, and serum amyloid A (SAA) is an acute-phase protein, which is upregulated by a variety of inflammatory stimuli (39). In IS patients who were treated with thrombolysis, the baseline (before thrombolysis treatment) SAP remained significantly and independently associated with 3-month death (40). Additionally, within the first 6 h after symptom onset, SAA had an AUC of 0.76 for predicting stroke-associated infections (41).

To sum up, SAP remained significantly and independently associated with 3-month death and can predict stroke-associated infections in hyperacute IS.

### Others Potential Biomarkers Associated With Inflammation and Oxidative Stress

Human β-defensin 2 (HBD-2) and chitotriosidase (ChT) are involved in the immune response and inflammation, and both are related to neurologic outcomes. Specifically, ChT is a sensitive parameter of macrophage activation and its elevated level in plasma reflects an inflammatory response (42). The baseline (pre tPA treatment) plasma ChT activity in IS patients was shown to be a short-term (i.e., at 48 h after stroke onset) predictor of tPA treatment outcome (43). HBD-2 mainly acts as an antimicrobial peptide and chemoattractant (44); baseline plasma HBD-2 level (within 4.5 h after symptom onset) was linked to neurologic decline at 24 and 48 h after stroke onset (33).

Retinol-binding protein 4 (RBP4), an adipokine primarily secreted by adipocytes and hepatocytes, has been implicated in oxidative stress, inflammation, and coronary artery calcification (45). Plasma RBP4 is a promising biomarker for distinguishing IS from HS patients during the hyperacute phase (within 6 h after symptom onset) as it was detected at a higher level in the former group (46). Moreover, the combination of RPB4 and GFAP improved the detection of IS (46). A recent clinical study reported that plasma RBP4 within 4.5 h after symptom onset was higher in IS patients than in HS patients (23).

Fluorescent molecular peroxidation products (FMPPs) are potential biomarkers of molecular oxidative damage that can increase the permeability of the BBB (47). Plasma FMPP level in IS patients before thrombolytic therapy predicted early neurologic deterioration at 48 h after symptom onset and was related to the occurrence of symptomatic HS after thrombolytic treatment of IS (47).

Adenosine acts as a vasodilator, inhibits inflammation, and might be neuroprotective (48). Within 3.3 h after symptom onset, serum adenosine showed a high value in separating IS from SM (49). In conclusion, RBP4 is a promising biomarker for distinguishing IS from HS patients during the hyperacute phase; and HBD-2 is a potential biomarker in assess prognosis of hyperacute IS. Additionally, ChT and FMPPs were predictors of tPA treatment outcome. And adenosine is a promising biomarker for distinguishing IS from SM patients during the hyperacute phase.

## Potential Biomarkers Associated with Vascular Injury and Angiogenesis

### Matrix Metalloproteinase-9 (MMP-9)

Matrix metalloproteinase-9 (MMP-9) is responsible for degradation of type IV collagen, laminin, and fibronectin, which are the major components of the basal lamina; and the loss of integrity of the basal lamina is considered to be the primary cause of edema after focal cerebral ischemia and hemorrhagic transformation (50). In IS patients within 6 h after symptom onset, plasma MMP-9 level substantially increased and correlated with the severity of the disease and infarct volume (51). Additionally, the baseline (pre tPA treatment) plasma MMP-9 level in IS patients predicted parenchymal hematoma (PH) appearance after tPA treatment (52). Another study showed that serum MMP-9 level ≥140 μg/l within 3 h after stroke onset and before tPA therapy predicted the occurrence of PH after therapy with a sensitivity of 92.0% and specificity of 74.0% (53).

In short, MMP-9 is a potential biomarker to evaluate the severity of hyperacute IS and predict the complications after tPA treatment.

### Metabolites of the l-Arginine Pathway

Nitric oxide (NO) is critical for the maintenance of vascular integrity (54). Asymmetric dimethylarginine (ADMA) and symmetric dimethylarginine (SDMA) are protein degradation products of l-arginine that can reduce NO production through direct or indirect pathways (55, 56). The levels of serum metabolites of the l-arginine pathway including l-arginine, ADMA, and SDMA were elevated in IS within 6 h after symptom onset compared to asymptomatic carotid stenosis, indicating a greater degree of endothelial dysfunction (57). Meanwhile, the serum concentration of l-arginine and ADMA/SDMA ratio were correlated with thrombo-inflammation within 6 h after IS onset (58); these correlations were in turn independently associated with risk of post-stroke infection but not other outcomes (58). Additionally, within 3.3 h after symptom onset, serum ADMA and SDMA showed a high value in separating IS from SM (49).

In conclusion, the different expression of metabolites of the l-arginine pathway in IS control and asymptomatic carotid stenosis patients prompts it as a potential biomarker in the diagnosis and assessment of the prognosis of hyperacute IS.

### Cellular Fibronectin (c-Fn)

Fibronectin exists in two forms: plasma fibronectin (p-Fn), which is primarily produced by hepatocytes; and c-Fn, which is mainly synthesized by endothelial cells. A high plasma level of c-Fn is indicative of endothelial damage (59). Plasma c-Fn level before tPA therapy was independently associated with tPA-induced hemorrhagic transformation (HT) (59), whereas serum c-Fn level ≥3.6 mg/l within 3 h after stroke onset and before tPA therapy predicted PH after tPA therapy with a sensitivity of 100.0%, specificity of 60.0%, and negative predictive value of 100.0% (53). Therefore, c-Fn is a potential biomarker to predict the complications after tPA treatment.

### Endostatin

Endostatin, an inhibitor of endothelial cell proliferation and migration, is derived from collagen XVIII, which is a major constituent of both endothelial and epithelial basement membranes (60). _High plasma endostatin level (within 3 h after symptom onset) was found to reflect an acute antiangiogenic status and predicted worse long-term functional outcome in IS patients (61). Another study reported that a plasma endostatin concentration higher than 1.203 ng/ml within 6 h after symptom onset could predict the development of epilepsy after a hyperacute stroke event (include IS and HS) (29). A recent clinical study reported that plasma endostatin within 4.5 h after symptom onset was higher in IS patients than in HS patients (23). As mentioned above, endostatin may be a potential biomarker in assessing the prognosis and complications of hyperacute IS.

### Other Potential Biomarkers Associated With Vascular Injury and Angiogenesis

Caveolin-1, which is highly expressed in endothelial cells, is an important regulator of endothelial permeability after cerebral ischemia (62). A low serum level of caveolin-1 (within 4.5 h after stroke onset) was associated with symptomatic HT after recombinant tPA therapy (63).

B-type natriuretic peptide (BNP), a neurohormone produced by the heart ventricles and the brain, promotes natriuresis and diuresis in the body, acting as a vasodilator with countering vasoconstrictor effects (64). Two fragments after the cleavage of a propeptide include N-terminal proBNP (NT-proBNP) which does not have biological activity, and formed-BNP which is biologically active (64). A recent clinical study reported that plasma NT-proBNP within 4.5 h after symptom onset was higher in IS patients than in HS patients (23).

Therefore, caveolin-1 is a potential biomarker to predict complications after tPA treatment. And NT-proBNP is a promising biomarker for distinguishing IS from HS patients during the hyperacute phase.

## Potential Genetic and Genomic Biomarkers

Epigenetic analysis and gene profiling have been used as diagnostic tools in cardiovascular diseases and cancer, and are increasingly being applied to stroke.

### RNAs

MicroRNAs (miRNAs) are a group of small, non-coding, endogenous single-stranded RNA molecules that are the main regulators of homeostasis in neurons; their dysregulation has been linked to specific pathologic processes in the brain (65, 66). In rats, miR-223-3p was upregulated whereas let-7b-3p was downregulated in the blood at 4 h after permanent MCAO-induced cerebral ischemia (67); and in patients with IS, circulating serum miRNA-221-3p and miRNA-382-5p levels were lower than in HCs within 6 h after symptom onset (68). Additionally, within 6 h after symptom onset, plasma miR-16 had an AUC of 0.775 for differentiating IS, with a sensitivity of 69.7% and specificity of 87.0%; and miR-16 AUC, sensitivity, and specificity in IS patients reached 0.95, 100.0, and 91.3% in stroke derived from large artery atherosclerosis (69). Moreover, plasma miR-16 level was higher in the poor prognosis (mRS 3–6) group than in the good prognosis (mRS 0–2) group (69). Another study showed that the levels of plasma miR-125a-5p, miR-125b-5p, and miR-143-3p were upregulated in IS within 6 h after symptom onset compared to HCs; and the set of plasma miRNAs (miR-125a-5p, miR-125b-5p, miR-143-3p) had an AUC of 0.90 for differentiating IS from HCs, with a sensitivity of 85.6% and specificity of 76.3%, which was superior to multimodal cranial computed tomography obtained for routine diagnostics (sensitivity: 72.5%) (70).

Circular RNAs (circRNAs) are a class of RNA molecules that may hold the key to understanding and properly manipulating tightly regulated gene expression patterns, as they are more highly expressed in brain tissues than in other tissues (71, 72). The levels of blood circPHKA2 (hsa _circ_0090002) and circBBS2 (hsa_circ_0039457) were downregulated in IS within 6 h after symptom onset compared to control subjects (73).

Transfer RNAs (tRNAs) are best known for their role in protein synthesis, tRNAs and tRNA-derived fragments (tRFs) also play a role in regulatory processes such as gene expression and translational control (74). Within 6 h after symptom onset, plasma tRFs had an AUC of 0.986 for differentiating HS from IS and SM (75).

Long non-coding RNAs (lncRNAs) represent an extensive, largely unexplored functional component of the genome, with multiple studies demonstrating that the brain expresses the highest amounts of lncRNAs among all tissue types, H19 is one of the best characterized lncRNA genes (76). A study showed that the levels of plasma H19 were significantly higher in IS patients within 3 h after symptom onset compared to HCs; and the plasma H19 had an AUC of 0.910 for differentiating IS from HCs, with a sensitivity of 80.6% and specificity of 92.0% (77).

As mentioned above, the different expression of RNAs in IS, HS, and SM suggests that it may be a potential biomarker in the diagnosis of hyperacute IS.

### Genetic Analysis and Gene Profiling

In a case–case prospective study, CD40-1C>T polymorphism (rs1883832) in peripheral blood was found to be associated with brain vessel re-occlusion after fibrinolysis in the early phase (within 3 h) after stroke onset (78). It was also reported that a large number of genes showed altered expression in the peripheral blood of humans as early as 3 h after IS onset, which was mainly attributable to neutrophils and was thought to contribute to tissue damage after stroke (79). Gene expression profiles in the peripheral blood (within 3 and 5 h after stroke onset) differ after cardioembolic compared with large-vessel atherosclerotic stroke (80). More specifically, genes showing altered expression in large-vessel atherosclerotic stroke were associated with platelets and monocytes and are known to be involved in the modulation of hemostasis, whereas those that were altered in cardioembolic stroke were expressed in neutrophils and related to the immune response to infection (80).

In short, CD40-1C>T polymorphism (rs1883832) can predict brain vessel re-occlusion after fibrinolysis after stroke onset; and genes may be potential biomarkers in assessing the complications of hyperacute IS.

## Potential Biomarkers Associated with Coagulation, Thrombosis, and Metabolism

Mean platelet volume (MPV), an index of platelet size, is related to thrombus formation and propagation and may contribute to acute thrombotic events (81). Disabling or fatal IS in thrombolytic patients was associated with high blood MPV level before initiation of recombinant tPA treatment (82).

Apolipoprotein CI and CIII (ApoC-I and ApoC-III, respectively) are involved in triglyceride metabolism. Plasma concentrations of ApoC-I and ApoC-III were lower in HS patients than in IS patients within 6 h after symptom onset (83).

Branched-chain amino acids (BCAAs) including valine, leucine, and isoleucine were reduced in rat plasma within 2 h after IS onset (84). A similar decrease in BCAAs was observed in human plasma within 6 ± 2 h after IS onset, and the degree of reduction was correlated with worse neurologic outcome (84).

A mass spectrometry-based proteomic analysis revealed that within 4.5 h after symptom onset, gelsolin, dihydropyrimidinase-related protein 2 (DPYSL2), and cystatin A in peripheral blood were independent predictors of poor outcome in IS (85).

Pregnenolone sulfate is one of the circulating metabolites. Within 3.3 h after symptom onset, serum pregnenolone sulfate had a high value in differentiating IS from SM (49).

As described above, MPV is a potential biomarker to predict the complications after tPA treatment. ApoC-I and ApoC-III, valine, leucine, isoleucine, gelsolin, DPYSL2, cystatin A, and pregnenolone sulfate are potential biomarkers to diagnose and assess the prognosis of hyperacute IS.

## Discussion

Stroke is a serious global public health problem. Multimodal neuroimaging is the standard method used for the diagnosis of hyperacute IS. However, it cannot detect changes in specific molecules and cell types in the brain, which is valuable for assessing etiology and prognosis. The pathophysiological mechanism of cerebral ischemia plays an important role in specifying the prevention and treatment of ischemic stroke, so the blood-based biomarkers in this manuscript are classified according to the pathophysiological mechanism of IS. Specifically, IS initiates a wide range of events called ischemic cascades at the beginning of cerebral ischemia. Ischemic events begin with gradual or sudden cerebral hypoperfusion, including cellular bioenergy failure, excitotoxicity, oxidative stress, blood-brain barrier dysfunction, microvascular injury, hemostatic activation, inflammation, and eventual neuronal, glial, and endothelial cell necrosis (86). Blood-based biomarkers can provide important information on clinical status beyond that obtained by standard tests and diagnostic procedures. Further, we list the main research results of the relevant cited literature in Supplementary Table 1, which includes biomarkers and classification, participants, sample type, onset to measurement time, major outcomes, association with stroke, study design, and references.

Delays in treatment greatly increase the disability and mortality rates of IS. Although a standardized stroke management protocol can shorten DNT and DPT, there is an inevitable delay between the time from symptom onset to arrival at the emergency room during which medical interventions cannot be administered. There are no methods for rapid detection of IS, which is mainly based on the observation of symptoms. Moreover, CT and MRI equipment are not widely installed in ambulances in China. Therefore, new tools for detecting IS outside of the hospital setting that are easy to operate without specialized knowledge and yield rapid results are needed. Blood-based biomarkers can serve as a foundation for the development of such diagnostic tests.

As an important part of the cytoskeleton of neurons, NfL has been proven to be associated with a variety of nervous system diseases, including Alzheimer's disease (AD), multiple sclerosis (MS), amyotrophic lateral sclerosis (ALS), Parkinson's disease (PD), and frontotemporal dementia (FTD), etc. It is conducive to predict, diagnose, monitor the progress, and evaluate the efficacy and prognosis of these diseases. Of note, NfL and cerebrovascular-related diseases (e.g., stroke, subarachnoid hemorrhage, traumatic brain injury, etc.) have also become research hotspots in recent years. Three important clinical studies also showed the associations of NfL with stroke. Specifically, one of these important studies showed that higher plasma NfL levels was independently associated with 3- or 6-month functional disability and higher all-cause mortality (87). Meanwhile, another study showed that serum NfL levels increased with the grade of age-related white matter changes and were able to predict unfavorable clinical outcome 90 days after stroke (88). Additionally, it is shown that serum NfL levels 7 days post-stroke independently predicted mRS 3 months post-stroke (89). In this clinical research, NfL was expected to objectively be one of the most promising surrogate markers for evaluating the clinical outcome of stroke. However, in the three studies, the authors did not state clearly that the blood sampling time was within 6 h after ischemic stroke onset (20 d, 24 h, 24 h, respectively), which was why we did not cite and summarize them before. Thus, we eagerly anticipate that more NfL studies will emerge in the future to better confirm the clinical value of blood-borne biomarkers in stroke.

Biomarkers are physiologic features or biological substances that can be objectively and reproducibly measured. The ideal biomarker has the following characteristics (25, 90): (1) accurate and reproducible; (2) measured in a standardized fashion; (3) acceptable to the patient and easy to interpret by clinicians; (4) non-invasive and inexpensive; (5) has sensitivity and specificity of at least 80%; (6) explains a reasonable proportion of the outcome independent of established predictors, with consistency across multiple studies; and (7) there is evidence that clinical management strategies can be determined based on biomarker level.

Some blood-based biomarkers have been identified for IS diagnosis, differentiation, and evaluation of severity and prognosis. However, there are several limitations associated with their use in clinical practice. Firstly, alterations in the brain may not be reflected by blood-based biomarkers because of the presence of the BBB. Secondly, IS is a heterogeneous clinical syndrome with various etiologies and complex clinical manifestations that cannot be represented by a single biomarker. Thirdly, blood-based biomarkers can be influenced by comorbidities such as hypertension and diabetes. Fourthly, most data on potential biomarkers of hyperacute IS have come from single-center studies with small samples in which candidate biomarker levels varied. Finally, a standard set of criteria for biomarker cutoff values is currently lacking.

In summary, blood-based biomarker testing can serve as a valuable adjunct to routine clinical examinations and neuroimaging in the diagnosis of hyperacute IS. Further exploration of the relationship between these biomarkers and the pathophysiology of hyperacute IS can guide treatment selection and facilitate prognostic assessment of hyperacute IS patients, thereby improving clinical outcomes.

## Author Contributions

YX and XW contributed to the conception and organization of the article. ZL and CY contributed to the literature search and manuscript writing. All authors contributed to the article and approved the submitted version.

## Conflict of Interest

The authors declare that the research was conducted in the absence of any commercial or financial relationships that could be construed as a potential conflict of interest.

## References

[1] Ekker MS Verhoeven JI Vaartjes I Jolink WMT Klijn CJM de Leeuw FE. Association of stroke among adults aged 18 to 49 years with long-term mortality. JAMA. (2019) 321:2113–23. 10.1001/jama.2019.6560 31121602 PMC6547225

[2] Wang W Jiang B Sun H Ru X Sun D Wang L. Prevalence, incidence, and mortality of stroke in china: results from a nationwide population-based survey of 480 687 adults. Circulation. (2017) 135:759–71. 10.1161/CIRCULATIONAHA.116.025250 28052979

[3] Powers WJ Rabinstein AA Ackerson T Adeoye OM Bambakidis NC Becker K. Guidelines for the early management of patients with acute ischemic stroke: 2019 update to the 2018 guidelines for the early management of acute ischemic stroke: a guideline for healthcare professionals from the American heart association/American stroke association. Stroke. (2019) 50:e344–418. 10.1161/STR.0000000000000211 31662037

[4] Peter R Korfiatis P Blezek D Oscar Beitia A Stepan-Buksakowska I Horinek D. A quantitative symmetry-based analysis of hyperacute ischemic stroke lesions in noncontrast computed tomography. Med Phys. (2017) 44:192–9. 10.1002/mp.12015 28066898 PMC5339891

[5] Kim SH Saver JL. Initial body temperature in ischemic stroke: nonpotentiation of tissue-type plasminogen activator benefit and inverse association with severity. Stroke. (2015) 46:132–6. 10.1161/STROKEAHA.114.006107 25424482 PMC4300232

[6] Nogueira RG Jadhav AP Haussen DC Bonafe A Budzik RF Bhuva P. Thrombectomy 6 to 24 hours after stroke with a mismatch between deficit and infarct. N Engl J Med. (2018) 378:11–21. 10.1056/NEJMoa1706442 29129157

[7] Albers GW Marks MP Kemp S Christensen S Tsai JP Ortega-Gutierrez S. Thrombectomy for stroke at 6 to 16 hours with selection by perfusion imaging. N Engl J Med. (2018) 378:708–18. 10.1056/NEJMoa1713973 29364767 PMC6590673

[8] Nowinski WL Gupta V Qian G He J Poh LE Ambrosius W. Automatic detection, localization, and volume estimation of ischemic infarcts in noncontrast computed tomographic scans: method and preliminary results. Invest Radiol. (2013) 48:661–70. 10.1097/RLI.0b013e31828d8403 23666092

[9] Papa L Akinyi L Liu MC Pineda JA Tepas JJ 3rd Oli MW. Ubiquitin C-terminal hydrolase is a novel biomarker in humans for severe traumatic brain injury. Crit Care Med. (2010) 38:138–44. 10.1097/CCM.0b013e3181b788ab 19726976 PMC3445330

[10] Rojo DR Prough DS Falduto MT Boone DR Micci MA Kahrig KM. Influence of stochastic gene expression on the cell survival rheostat after traumatic brain injury. PLoS ONE. (2011) 6:e23111. 10.1371/journal.pone.0023111 21853077 PMC3154935

[11] Reinicke AT Laban K Sachs M Kraus V Walden M Damme M. Ubiquitin C-terminal hydrolase L1 (UCH-L1) loss causes neurodegeneration by altering protein turnover in the first postnatal weeks. Proc Natl Acad Sci USA. (2019) 116:7963–72. 10.1073/pnas.1812413116 30923110 PMC6475369

[12] Ren C Zoltewicz S Guingab-Cagmat J Anagli J Gao M Hafeez A. Different expression of ubiquitin C-terminal hydrolase-L1 and αII-spectrin in ischemic and hemorrhagic stroke: Potential biomarkers in diagnosis. Brain Res. (2013) 1540:84–91. 10.1016/j.brainres.2013.09.051 24140110

[13] Liu MC Akinyi L Scharf D Mo J Larner SF Muller U. Ubiquitin C-terminal hydrolase-L1 as a biomarker for ischemic and traumatic brain injury in rats. Eur J Neurosci. (2010) 31:722–32. 10.1111/j.1460-9568.2010.07097.x 20384815 PMC2855688

[14] Luger S Jæger HS Dixon J Bohmann FO Schaefer J Richieri SP. Diagnostic accuracy of glial fibrillary acidic protein and ubiquitin carboxy-terminal hydrolase-l1 serum concentrations for differentiating acute intracerebral hemorrhage from ischemic stroke. Neurocrit Care. (2020) 33:39–48. 10.1007/s12028-020-00931-5 32096121

[15] Ren C Kobeissy F Alawieh A Li N Li N Zibara K. Assessment of serum UCH-L1 and GFAP in acute stroke patients. Sci Rep. (2016) 6:24588. 10.1038/srep24588 27074724 PMC4830936

[16] Dambinova SA Khounteev GA Izykenova GA Zavolokov IG Ilyukhina AY Skoromets AA. Blood test detecting autoantibodies to N-methyl-D-aspartate neuroreceptors for evaluation of patients with transient ischemic attack and stroke. Clin Chem. (2003) 49:1752–62. 10.1373/49.10.1752 14500616

[17] Khalil M Teunissen CE Otto M Piehl F Sormani MP Gattringer T. Neurofilaments as biomarkers in neurological disorders. Nat Rev Neurol. (2018) 14:577–89. 10.1038/s41582-018-0058-z 30171200

[18] De Marchis GM Katan M Barro C Fladt J Traenka C Seiffge DJ. Serum neurofilament light chain in patients with acute cerebrovascular events. Eur J Neurol. (2018) 25:562–8. 10.1111/ene.13554 29281157

[19] Eng LF Ghirnikar RS Lee YL. Glial fibrillary acidic protein: GFAP-thirty-one years (1969-2000). Neurochem Res. (2000) 25:1439–51. 10.1023/A:1007677003387 11059815

[20] Dvorak F Haberer I Sitzer M Foerch C. Characterisation of the diagnostic window of serum glial fibrillary acidic protein for the differentiation of intracerebral haemorrhage and ischaemic stroke. Cerebrovasc Dis. (2009) 27:37–41. 10.1159/000172632 19018136

[21] Foerch C Niessner M Back T Bauerle M De Marchis GM Ferbert A. Diagnostic accuracy of plasma glial fibrillary acidic protein for differentiating intracerebral hemorrhage and cerebral ischemia in patients with symptoms of acute stroke. Clin Chem. (2012) 58:237–45. 10.1373/clinchem.2011.172676 22125303

[22] Luger S Witsch J Dietz A Hamann GF Minnerup J Schneider H. Glial fibrillary acidic protein serum levels distinguish between intracerebral hemorrhage and cerebral ischemia in the early phase of stroke. Clin Chem. (2017) 63:377–85. 10.1373/clinchem.2016.263335 27881450

[23] Bustamante A Penalba A Orset C Azurmendi L Llombart V Simats A. Blood biomarkers to differentiate ischemic and hemorrhagic strokes. Neurology. (2021) 96:e1928–39. 10.1212/WNL.0000000000011742 33674361

[24] Barger SW Van Eldik LJ. S100 beta stimulates calcium fluxes in glial and neuronal cells. J Biol Chem. (1992) 267:9689–94. 10.1016/S0021-9258(19)50145-4 1315768

[25] Makris K Haliassos A Chondrogianni M Tsivgoulis G. Blood biomarkers in ischemic stroke: potential role and challenges in clinical practice and research. Crit Rev Clin Lab Sci. (2018) 55:294–328. 10.1080/10408363.2018.1461190 29668333

[26] Choi JI Ha SK Lim DJ Kim SD Kim SH. S100ss, matrix metalloproteinase-9, D-dimer, and heat shock protein 70 are serologic biomarkers of acute cerebral infarction in a mouse model of transient MCA occlusion. J Korean Neurosurg Soc. (2018) 61:548–58. 10.3340/jkns.2017.0200 29724092 PMC6129755

[27] Zhou S Bao J Wang Y Pan S. S100beta as a biomarker for differential diagnosis of intracerebral hemorrhage and ischemic stroke. Neurol Res. (2016) 38:327–32. 10.1080/01616412.2016.1152675 27078704

[28] Pusch G Debrabant B Molnar T Feher G Papp V Banati M. Early dynamics of p-selectin and interleukin 6 predicts outcomes in ischemic stroke. J Stroke Cerebrovasc Dis. (2015) 24:1938–47. 10.1016/j.jstrokecerebrovasdis.2015.05.005 26051664

[29] Abraira L Santamarina E Cazorla S Bustamante A Quintana M Toledo M. Blood biomarkers predictive of epilepsy after an acute stroke event. Epilepsia. (2020) 61:2244–53. 10.1111/epi.16648 32857458

[30] Tedgui A Mallat Z. Anti-inflammatory mechanisms in the vascular wall. Circ Res. (2001) 88:877–87. 10.1161/hh0901.090440 11348996

[31] Ohara J Paul WE. Receptors for B-cell stimulatory factor-1 expressed on cells of haematopoietic lineage. Nature. (1987) 325:537–40. 10.1038/325537a0 3100961

[32] Lowenthal JW Castle BE Christiansen J Schreurs J Rennick D Arai N. Expression of high affinity receptors for murine interleukin 4 (BSF-1) on hemopoietic and nonhemopoietic cells. J Immunol. (1988) 140:456–64. 2961813

[33] García-Berrocoso T Giralt D Bustamante A Llombart V Rubiera M Penalba A. Role of beta-defensin 2 and interleukin-4 receptor as stroke outcome biomarkers. J Neurochem. (2014) 129:463–72. 10.1111/jnc.12649 24386991

[34] Rodríguez-Yáñez M Castellanos M Sobrino T Brea D Ramos-Cabrer P Pedraza S. Interleukin-10 facilitates the selection of patients for systemic thrombolysis. BMC Neurol. (2013) 13:62. 10.1186/1471-2377-13-62 23773291 PMC3710209

[35] Zhu B Pan Y Jing J Meng X Zhao X Liu L. Neutrophil counts, neutrophil ratio, and new stroke in minor ischemic stroke or TIA. Neurology. (2018) 90:e1870–8. 10.1212/WNL.0000000000005554 29678934

[36] Lux D Alakbarzade V Bridge L Clark CN Clarke B Zhang L. The association of neutrophil-lymphocyte ratio and lymphocyte-monocyte ratio with 3-month clinical outcome after mechanical thrombectomy following stroke. J Neuroinflamm. (2020) 17:60. 10.1186/s12974-020-01739-y 32070366 PMC7026966

[37] Gong P Liu Y Gong Y Chen G Zhang X Wang S. The association of neutrophil to lymphocyte ratio, platelet to lymphocyte ratio, and lymphocyte to monocyte ratio with post-thrombolysis early neurological outcomes in patients with acute ischemic stroke. J Neuroinflamm. (2021) 18:51. 10.1186/s12974-021-02090-6 33610168 PMC7896410

[38] Maestrini I Tagzirt M Gautier S Dupont A Mendyk AM Susen S. Analysis of the association of MPO and MMP-9 with stroke severity and outcome: cohort study. Neurology. (2020) 95:e97–108. 10.1212/WNL.0000000000009179 32111692

[39] Ray A Shakya A Kumar D Benson MD Ray BK. Inflammation-responsive transcription factor SAF-1 activity is linked to the development of amyloid A amyloidosis. J Immunol. (2006) 177:2601–9. 10.4049/jimmunol.177.4.2601 16888022

[40] Gori AM Giusti B Piccardi B Nencini P Palumbo V Nesi M. Inflammatory and metalloproteinases profiles predict three-month poor outcomes in ischemic stroke treated with thrombolysis. J Cereb Blood Flow Metab. (2017) 37:3253–61. 10.1177/0271678X17695572 28266892 PMC5584701

[41] Schweizer J Bustamante A Lapierre-Fétaud V Faura J Scherrer N Azurmendi Gil L. SAA (Serum Amyloid A): a novel predictor of stroke-associated infections. Stroke. (2020) 51:3523–30. 10.1161/STROKEAHA.120.030064 33161846

[42] van Eijk M van Roomen CP Renkema GH Bussink AP Andrews L Blommaart EF. Characterization of human phagocyte-derived chitotriosidase, a component of innate immunity. Int Immunol. (2005) 17:1505–12. 10.1093/intimm/dxh328 16214810

[43] Bustamante A Dominguez C Rodriguez-Sureda V Vilches A Penalba A Giralt D. Prognostic value of plasma chitotriosidase activity in acute stroke patients. Int J Stroke. (2013) 9:910–6. 10.1111/ijs.12160 24148838

[44] Duits LA Ravensbergen B Rademaker M Hiemstra PS Nibbering PH. Expression of beta-defensin 1 and 2 mRNA by human monocytes, macrophages and dendritic cells. Immunology. (2002) 106:517–25. 10.1046/j.1365-2567.2002.01430.x 12153515 PMC1782759

[45] Zabetian-Targhi F Mahmoudi MJ Rezaei N Mahmoudi M. Retinol binding protein 4 in relation to diet, inflammation, immunity, and cardiovascular diseases. Adv Nutr. (2015) 6:748–62. 10.3945/an.115.008292 26567199 PMC4642414

[46] Llombart V Garcia-Berrocoso T Bustamante A Giralt D Rodriguez-Luna D Muchada M. Plasmatic retinol-binding protein 4 and glial fibrillary acidic protein as biomarkers to differentiate ischemic stroke and intracerebral hemorrhage. J Neurochem. (2016) 136:416–24. 10.1111/jnc.13419 26526443

[47] Llombart V Dominguez C Bustamante A Rodriguez-Sureda V Martín-Gallán P Vilches A. Fluorescent molecular peroxidation products. Stroke. (2014) 45:432–7. 10.1161/STROKEAHA.113.003431 24335228

[48] Chen JF Lee CF Chern Y. Adenosine receptor neurobiology: overview. Int Rev Neurobiol. (2014) 119:1–49. 10.1016/B978-0-12-801022-8.00001-5 25175959

[49] Tiedt S Brandmaier S Kollmeier H Duering M Artati A Adamski J. Circulating metabolites differentiate acute ischemic stroke from stroke mimics. Ann Neurol. (2020) 88:736–46. 10.1002/ana.25859 32748431

[50] Miao Y Liao JK. Potential serum biomarkers in the pathophysiological processes of stroke. Exp Rev Neurother. (2014) 14:173–85. 10.1586/14737175.2014.875471 24417214 PMC3939679

[51] Demir R Ulvi H Özel L Özdemir G Güzelcik M Aygül R. Relationship between plasma metalloproteinase-9 levels and volume and severity of infarct in patients with acute ischemic stroke. Acta Neurol Belgica. (2012) 112:351–6. 10.1007/s13760-012-0067-4 22581515

[52] Montaner J Molina CA Monasterio J Abilleira S Arenillas JF Ribó M. Matrix metalloproteinase-9 pretreatment level predicts intracranial hemorrhagic complications after thrombolysis in human stroke. Circulation. (2003) 107:598–603. 10.1161/01.CIR.0000046451.38849.90 12566373

[53] Castellanos M Sobrino Ts Millan Mn Garcia Ma Arenillas J Nombela F. Serum cellular fibronectin and matrix metalloproteinase-9 as screening biomarkers for the prediction of parenchymal hematoma after thrombolytic therapy in acute ischemic stroke. Stroke. (2007) 38:1855–9. 10.1161/STROKEAHA.106.481556 17478737

[54] Stühlinger MC Stanger O. Asymmetric dimethyl-L-arginine (ADMA): a possible link between homocyst(e)ine and endothelial dysfunction. Curr Drug Metab. (2005) 6:3–14. 10.2174/1389200052997393 15720202

[55] Nishiyama Y Ueda M Katsura K Otsuka T Abe A Nagayama H. Asymmetric dimethylarginine (ADMA) as a possible risk marker for ischemic stroke. J Neurol Sci. (2010) 290:12–5. 10.1016/j.jns.2009.12.020 20060545

[56] Sobczak A Goniewicz ML Szoltysek-Boldys I. ADMA and SDMA levels in healthy men exposed to tobacco smoke. Atherosclerosis. (2009) 205:357–9. 10.1016/j.atherosclerosis.2008.12.039 19181316 PMC2789676

[57] Molnar T Pusch G Papp V Feher G Szapary L Biri B. The L-arginine pathway in acute ischemic stroke and severe carotid stenosis: temporal profiles and association with biomarkers and outcome. J Stroke Cerebrovasc Dis. (2014) 23:2206–14. 10.1016/j.jstrokecerebrovasdis.2014.05.002 25018114

[58] Molnar T Pusch G Nagy L Keki S Berki T Illes Z. Correlation of the L-Arginine pathway with thrombo-inflammation may contribute to the outcome of acute ischemic stroke. J Stroke Cerebrovasc Dis. (2016) 25:2055–60. 10.1016/j.jstrokecerebrovasdis.2016.05.018 27263035

[59] Castellanos M Leira R Serena J Blanco M Pedraza S Castillo J. Plasma cellular-fibronectin concentration predicts hemorrhagic transformation after thrombolytic therapy in acute ischemic stroke. Stroke. (2004) 35:1671–6. 10.1161/01.STR.0000131656.47979.39 15166391

[60] Sudhakar A Sugimoto H Yang C Lively J Zeisberg M Kalluri R. Human tumstatin and human endostatin exhibit distinct antiangiogenic activities mediated by alpha v beta 3 and alpha 5 beta 1 integrins. Proc Natl Acad Sci USA. (2003) 100:4766–71. 10.1073/pnas.0730882100 12682293 PMC153630

[61] Navarro-Sobrino M Rosell A Hernández-Guillamon M Penalba A Boada C Domingues-Montanari S. A large screening of angiogenesis biomarkers and their association with neurological outcome after ischemic stroke. Atherosclerosis. (2011) 216:205–11. 10.1016/j.atherosclerosis.2011.01.030 21324462

[62] Xu L Guo R Xie Y Ma M Ye R Liu X. Caveolae: molecular insights and therapeutic targets for stroke. Exp Opin Ther Targets. (2015) 19:633–50. 10.1517/14728222.2015.1009446 25639269

[63] Castellanos M van Eendenburg C Gubern C Kadar E Huguet G Puig J. Low levels of caveolin-1 predict symptomatic bleeding after thrombolytic therapy in patients with acute ischemic stroke. Stroke. (2018) 49:1525–7. 10.1161/STROKEAHA.118.020683 29712879

[64] Ng GJL Quek AML Cheung C Arumugam TV Seet RCS. Stroke biomarkers in clinical practice: a critical appraisal. Neurochem Int. (2017) 107:11–22. 10.1016/j.neuint.2017.01.005 28088349

[65] Chan PH. Reactive oxygen radicals in signaling and damage in the ischemic brain. J Cereb Blood Flow Metab. (2001) 21:2–14. 10.1097/00004647-200101000-00002 11149664

[66] Wang X Chen S Ni J Cheng J Jia J Zhen X. miRNA-3473b contributes to neuroinflammation following cerebral ischemia. Cell Death Dis. (2018) 9:11. 10.1038/s41419-017-0014-7 29317607 PMC5849032

[67] Li S Chen L Zhou X Li J Liu J. miRNA-223-3p and let-7b-3p as potential blood biomarkers associated with the ischemic penumbra in rats. Acta Neurobiol Exp. (2019) 79:205–16. 10.21307/ane-2019-018 31342956

[68] Wang Y Ma Z Kan P Zhang B. The diagnostic value of serum miRNA-221-3p, miRNA-382-5p, and miRNA-4271 in ischemic stroke. J Stroke Cerebrovasc Dis. (2017) 26:1055–60. 10.1016/j.jstrokecerebrovasdis.2016.12.019 28111007

[69] Tian C Li Z Yang Z Huang Q Liu J Hong B. Plasma MicroRNA-16 is a biomarker for diagnosis, stratification, and prognosis of hyperacute cerebral infarction. PLoS ONE. (2016) 11:e0166688. 10.1371/journal.pone.0166688 27846323 PMC5112925

[70] Tiedt S Prestel M Malik R Schieferdecker N Duering M Kautzky V. RNA-Seq identifies circulating miR-125a-5p, miR-125b-5p, and miR-143-3p as potential biomarkers for acute ischemic stroke. Circ Res. (2017) 121:970–80. 10.1161/CIRCRESAHA.117.311572 28724745

[71] Ritchie ME Phipson B Wu D Hu Y Law CW Shi W. limma powers differential expression analyses for RNA-sequencing and microarray studies. Nucleic Acids Res. (2015) 43:e47. 10.1093/nar/gkv007 25605792 PMC4402510

[72] Zhang Y Xue W Li X Zhang J Chen S Zhang JL. The biogenesis of nascent circular RNAs. Cell Rep. (2016) 15:611–24. 10.1016/j.celrep.2016.03.058 27068474

[73] Lu D Ho ES Mai H Zang J Liu Y Li Y. Identification of blood circular RNAs as potential biomarkers for acute ischemic stroke. Front Neurosci. (2020) 14:81. 10.3389/fnins.2020.00081 32116524 PMC7015875

[74] Li S Xu Z Sheng J. tRNA-derived small RNA: a novel regulatory small non-coding RNA. Genes. (2018) 9:246. 10.3390/genes9050246 29748504 PMC5977186

[75] Nguyen TTM van der Bent ML Wermer MJH van den Wijngaard IR van Zwet EW de Groot B. Circulating tRNA fragments as a novel biomarker class to distinguish acute stroke subtypes. Int J Mol Sci. (2020) 22:135. 10.3390/ijms22010135 33374482 PMC7796003

[76] Derrien T Johnson R Bussotti G Tanzer A Djebali S Tilgner H. The GENCODE v7 catalog of human long noncoding RNAs: analysis of their gene structure, evolution, and expression. Genome Res. (2012) 22:1775–89. 10.1101/gr.132159.111 22955988 PMC3431493

[77] Wang J Zhao H Fan Z Li G Ma Q Tao Z. Long noncoding RNA H19 promotes neuroinflammation in ischemic stroke by driving histone deacetylase 1-Dependent M1 microglial polarization. Stroke. (2017) 48:2211–21. 10.1161/STROKEAHA.117.017387 28630232

[78] del Río-Espínola A Fernández-Cadenas I Rubiera M Quintana M Domingues-Montanari S Mendióroz M. CD40-1C>T polymorphism (rs1883832) is associated with brain vessel reocclusion after fibrinolysis in ischemic stroke. Pharmacogenomics. (2010) 11:763–72. 10.2217/pgs.10.44 20504251

[79] Tang Y Xu H Du X Lit L Walker W Lu A. Gene expression in blood changes rapidly in neutrophils and monocytes after ischemic stroke in humans: a microarray study. J Cereb Blood Flow Metab. (2006) 26:1089–102. 10.1038/sj.jcbfm.9600264 16395289

[80] Xu H Tang Y Liu DZ Ran R Ander BP Apperson M. Gene expression in peripheral blood differs after cardioembolic compared with large-vessel atherosclerotic stroke: biomarkers for the etiology of ischemic stroke. J Cereb Blood Flow Metab. (2008) 28:1320–8. 10.1038/jcbfm.2008.22 18382470

[81] Ross R. Atherosclerosis–an inflammatory disease. N Engl J Med. (1999) 340:115–26. 10.1056/NEJM199901143400207 9887164

[82] Staszewski J Pogoda A Data K Walczak K Nowocień M Frankowska E. The mean platelet volume on admission predicts unfavorable stroke outcomes in patients treated with IV thrombolysis. Clin Interve Aging. (2019) 14:493–503. 10.2147/CIA.S195451 30880930 PMC6398411

[83] Allard L Lescuyer P Burgess J Leung KY Ward M Walter N. ApoC-I and ApoC-III as potential plasmatic markers to distinguish between ischemic and hemorrhagic stroke. Proteomics. (2004) 4:2242–51. 10.1002/pmic.200300809 15274118

[84] Kimberly WT Wang Y Pham L Furie KL Gerszten RE. Metabolite profiling identifies a branched chain amino acid signature in acute cardioembolic stroke. Stroke. (2013) 44:1389–95. 10.1161/STROKEAHA.111.000397 23520238 PMC3816089

[85] García-Berrocoso T Penalba A Boada C Giralt D Cuadrado E Colomé N. From brain to blood: new biomarkers for ischemic stroke prognosis. J Proteom. (2013) 94:138–48. 10.1016/j.jprot.2013.09.005 24061000

[86] Saenger AK Christenson RH. Stroke biomarkers: progress and challenges for diagnosis, prognosis, differentiation, and treatment. Clin Chem. (2010) 56:21–33. 10.1373/clinchem.2009.133801 19926776

[87] Gendron TF Badi MK Heckman MG Jansen-West KR Vilanilam GK Johnson PW. Plasma neurofilament light predicts mortality in patients with stroke. Sci Transl Med. (2020) 12:eaay1913. 10.1126/scitranslmed.aay1913 33177179 PMC9534269

[88] Uphaus T Bittner S Gröschel S Steffen F Muthuraman M Wasser K. NfL (Neurofilament Light Chain) levels as a predictive marker for long-term outcome after ischemic stroke. Stroke. (2019) 50:3077–84. 10.1161/STROKEAHA.119.026410 31537188

[89] Tiedt S Duering M Barro C Kaya AG Boeck J Bode FJ. Serum neurofilament light: a biomarker of neuroaxonal injury after ischemic stroke. Neurology. (2018) 91:e1338–47. 10.1212/WNL.0000000000006282 30217937

[90] El Kadmiri N Said N Slassi I El Moutawakil B Nadifi S. Biomarkers for alzheimer disease: classical and novel candidates' review. Neuroscience. (2018) 370:181–90. 10.1016/j.neuroscience.2017.07.017 28729061

