# Supplementary material for: Blood-Based Biomarkers: A Forgotten Friend of Hyperacute Ischemic Stroke
Source: Front Neurol. 2021 Jun 8;12:634717. doi: 10.3389/fneur.2021.634717 (PMC8217611; doi:10.3389/fneur.2021.634717)
Supplement: Supplementary file 1 [file Table_1.DOCX]

**Supplemental Table 1.** **An overview of the major researches involved in blood-based biomarkers of hyperacute IS published currently.**

| **Type** | **Biomarker** | **Participants** | **Sample type** | **OTMT** | **Outcomes** | **Association** | **Study design** | **References** |  |
| --- | --- | --- | --- | --- | --- | --- | --- | --- | --- |
| Neuronal injury | UCH-L1 | sham (n=8)  Isch-3 h (n=8)  Isch-6 h (n=8)  Hem-3 h (n=8)  Hem-6 h (n=8) | Serum | 3 h, 6 h | Isch-3 h ↑ vs. sham  Isch-6 h ↑ vs. sham | N/A | Animal research | Brain Research, 2013 |  |
|  |  | naïve (n=5)  sham (n=5)  MCAO-30 min (n=9)  MCAO-2 h (n=13) | Serum | 6 h | MCAO-30 min ↑ vs. sham  MCAO-2 h ↑ vs. sham | N/A | Animal research | Eur J Neurosci, 2010 |  |
|  |  | HS (n=64)  IS (n=181)  SM (n=16) | Serum | <4 h  <6 h | HS ↑ vs. IS  HS ↑ vs. SM | The AUC of serum UCH-L1 for distinguishing HS patients from IS and SM patients were 0.590. | Prospective two-center study | Neurocritical care, 2020 |  |
|  |  | HS (n=45)  IS (n=79)  CTRL (n=57) | Serum | <4.5 h | CTRL < IS ↑< HS ↑ | The AUCs of serum UCH-L1 for distinguishing IS patients from controls and IS from HS patients were 0.64 and 0.62, respectively. | Case-control study | Sci Rep, 2016 |  |
|  | NMDAR | IS (n=31)  CTRL (n=230) | Serum | <3 h | IS ↑ vs. CTRL | Serum NMDAR had an AUC of 0.99 for diagnosing IS with a sensitivity of 97.0% and specificity of 98.0% using the best cutoff point of 2.0 μg/l. | Case-control study | Clin Chem, 2003 |  |
|  | NfL | IS (n=504)  TIA (n=111) | Serum | <3 h | IS ↑ vs. TIA | Serum NfL were associated with clinical severity on admission and TIA diagnosis. | Cohort study | Eur J Neurol, 2018 |  |
| Glial activation | GFAP | IS (n=45)  HS (n=18) | Serum | 2-6 h | HS ↑ vs. IS | Two hours after stroke onset, serum GFAP values were significantly correlated with HS volume. | Prospective study | Cerebrovasc Dis, 2009 |  |
|  |  | HS (n=39)  IS (n=163)  SM (n=3) | Plasma | <4.5 h | HS ↑ vs. IS | Plasma GFAP for differentiating HS from IS and SM was found to be 0.915, with a sensitivity of 84.2% and specificity of 96.3% using a cutoff value of 0.29 μg/l. | Prospective study | Clin Chem, 2012 |  |
|  |  | HS (n=45)  IS (n=79)  CTRL (n=57) | Serum | <4.5 h | CTRL < IS ↑< HS ↑ | Higher GFAP levels were associated with stroke severity and history of prior stroke. | Case-control study | Sci Rep, 2016 |  |
|  |  | HS (n=64)  IS (n=181)  SM (n=16) | Serum | <4 h  <6 h | HS ↑ vs. IS  HS ↑ vs. SM | Serum GFAP had an AUC of 0.866 for differentiating HS from IS and SM, with a sensitivity of 75.0% and specificity of 84.0% using an optimal cutoff point of 72 ng/l. | Prospective two-center study | Neurocritical care, 2020 |  |
|  |  | HS (n=45)  IS (n=146)  SM (n=11) | Serum | <6 h | HS ↑ vs. IS | Serum GFAP concentrations were positively correlated with HS volume. | Prospective study | Clin Chem, 2017 |  |
|  |  | HS (n=35)  IS (n=154) | Plasma | <4.5 h | HS ↑ vs. IS | A biomarker panel including RBP4, NT-proBNP and GFAP provided moderate but potentially useful sensitivity rates at 100% specificity for IS diagnosis. | Cohort study | Neurology, 2021 |  |
|  | S100β | sham (n=5), MCAO-30 min (n=10)  MCAO-60 min (n=10) | Plasma | 4 h | sham< MCAO-60 min ↑< MCAO-30 min ↑ | Plasma S100β expressions correlated with infarction volumes and neurological deficits. | Animal research | J Korean Neurosurg Soc, 2018 |  |
|  |  | HS (n=46)  IS (n=71) | Plasma | <6 h | HS ↑ vs. IS | Plasma S100β concentration was significantly elevated in patients with poor functional outcome vs. those with favorable functional outcome. | Prospective study | Neurol Res, 2016 |  |
|  |  | IS (n=76)  PD (n=66)  Significant carotid stenosis (n=44) | Serum | 6 h | N/A | A high serum concentration of S100β was associated with increased risk of poststroke infections. | Prospective study | Journal of Stroke and Cerebrovascular Diseases, 2015 |  |
|  |  | poststroke epilepsy (n=51)  non- poststroke epilepsy (n=844) | Plasma | <6 h | non- poststroke epilepsy ↑ vs. poststroke epilepsy | A plasma S100β concentration was lower than 1.364 pg/ml could predict the development of epilepsy after a hyperacute stroke event (include IS and HS). | Prospective longitudinal study | Epilepsia, 2020 |  |
| Inflammation and oxidative stress | IL-10 | 184 IS patients received tPA treatment, Poor outcome (n=81)  Good outcome (n=103) | Serum | Pre tPA treatment | Poor outcome vs. Good outcome ↑ | A serum IL-10 level ≥30 pg/ml predicted a favorable functional outcome after tPA treatment at 3 months with a sensitivity of 86.0% and specificity of 88.0%. | Prospective study | BMC neurology, 2013 |  |
|  | IL-4R | Worsening 24 h (n=17)  non-Worsening 24 h (n=62)  Worsening 48 h (n=21)  non-Worsening 48 h (n=45) | Plasma | <4.5 h | Worsening 24 h ↑ vs. non-Worsening 24 h  Worsening 48 h ↑ vs. non-Worsening 48 h | Plasma IL-4R level was an independent predictor of poor neurologic prognosis with a sensitivity of 53.0% and specificity of 72.0% at 24 h and a sensitivity of 52.0% and specificity of 73.0% at 48 h after stroke onset using the same cutoff point of 503.40 ng/l. | Prospective study | Journal of Neurochemistry, 2014 |  |
|  | NLR, PLR | END group (n=193)  ENI group (n=398) | Blood | Pre tPA treatment | END group ↑ vs. ENI group | The AUCs of NLR and PLR for predicting post-thrombolysis END and NLR predicting post-thrombolysis ENI were 0.763, 0.703, and 0.695, respectively. | Prospective study | J Neuroinflammation, 2021 |  |
|  | Neutrophil, NLR | mRS 0–1 (n=130)  non-mRS 0–1 (n=125) | Serum | <4.5 h | mRS 0–1 vs. non-mRS 0–1 ↑ | Higher neutrophil counts and NLR were independently associated with worse outcomes and higher mortality rates at month 3. | Prospective study | Neurology, 2020 |  |
|  | SAP | 327 patients with IS treated with intravenous thrombolys | Serum | Pre tPA treatment | N/A | The baseline (before thrombolysis treatment) SAP remained significantly and independently associated with three-month death. | Prospective study | J Cereb Blood Flow Metab, 2017 |  |
|  |  | 367 patients with IS | Serum | <6 h | N/A | SAA had an AUC of 0.76 for predicting stroke-associated infections. | Prospective study | Stroke, 2020 |  |
|  | HBD-2 | Worsening 24 h (n=17)  Non-Worsening 24 h (n=62)  Worsening 48 h (n=21)  non-Worsening 48 h (n=45) | Plasma | <4.5 h | Worsening 24 h ↑ vs. non-Worsening 24 h  Worsening 48 h ↑ vs. non-Worsening 48 h | Baseline plasma HBD-2 level (<4.5 h after stroke onset) was linked to neurologic decline at 24 and 48 h after stroke onset. | Prospective study | Journal of Neurochemistry, 2014 |  |
|  | ChT | IS (n=159)  CTRL (n=51) | Plasma | Pre tPA treatment | IS ↑ vs. CTRL | Plasma ChT activity in IS patients was shown to be a short-term (ie, at 48 h after stroke onset) predictor of tPA treatment outcome. | Prospective study | International Journal of Stroke, 2013 |  |
|  | RBP4 | IS (n=38)  HS (n=28) | Plasma | <6 h | IS ↑ vs. HS | Plasma RBP4 is a promising biomarker for distinguishing IS from HS patients, and the combination of RPB4 and GFAP can improve the detection of IS. | Cohort study | J Neurochem, 2016 |  |
|  |  | HS (n=35)  IS (n=154) | Plasma | <4.5 h | IS ↑ vs. HS | A biomarker panel including RBP4, NT-proBNP and GFAP provided moderate but potentially useful sensitivity rates at 100% specificity for IS diagnosis. | Cohort study | Neurology, 2021 |  |
|  | FMPPs | Worsening at 48 h (n=16)  Non-Worsening at 48 h (n=164) | Plasma | Pre tPA treatment | Worsening at 48 h ↑ vs. non-Worsening at 48 h | Plasma FMPP level predicted early neurologic deterioration at 48 h after symptom onset and was related to the occurrence of symptomatic HS after thrombolytic treatment of IS. | Prospective study | Stroke, 2014 |  |
|  | Adenosine | IS (n=508)  SM (n=349) | Serum | <3.3 h | N/A | Four metabolites including adenosine outperform CT to differentiate IS and SM. | Prospective study | Ann Neurol, 2020 |  |
| Vascular injury and angiogenesis | MMP-9 | IS (n=32)  CTRL (n=30) | Plasma | <6 h | IS ↑ vs. CTRL | Plasma MMP-9 level correlated with the severity of the disease and infarct volume. | Case-control  study | Acta Neurol Belg, 2012 |  |
|  |  | PH (n=5)  non-PH (n=36) | Plasma | Pre tPA treatment | PH ↑ vs. non-PH | N/A | Prospective study | Circulation, 2003 |  |
|  |  | PH (n=12)  non-PH (n=122) | Serum | <3 h | PH ↑ vs. non-PH | Serum MMP-9 level ≥140 μg/l within 3 h after stroke onset and before tPA therapy predicted the occurrence of PH after therapy with a sensitivity of 92.0% and specificity of 74.0%. | Prospective study | Stroke, 2007 |  |
|  | l-arginine, ADMA, SDMA | IS (n=55)  AsCS (n=44)  CTRL (n=45) | Serum | <6 h | IS ↑ vs. AsCS  IS ↑ vs. CTRL | N/A | Case-control  study | Journal of Stroke and Cerebrovascular Diseases, 2014 |  |
|  | l-arginine, ADMA/  SDMA | 55 patients with IS | Serum | <6 h | N/A | l-arginine and ADMA/SDMA ratio were correlated with thromboinflammation; these correlations were in turn independently associated with risk of poststroke infection. | Prospective study | J Stroke Cerebrovasc Dis, 2016 |  |
|  | ADMA, SDMA | IS (n=508)  SM (n=349) | Serum | <3.3 h | N/A | Four metabolites including SDMA and ADMA outperform CT to differentiate IS and SM. | Prospective study | Ann Neurol, 2020 |  |
|  | c-Fn | HT (n=26)  Non-HT (n=61)  CTRL (n=30) | Plasma | Pre tPA treatment | HT ↑ vs. non-HT  HT ↑ vs. CTRL | Plasma c-Fn level before tPA therapy was independently associated with tPA-induced HT. | Prospective study | Stroke, 2004 |  |
|  |  | PH (n=12)  Non-PH (n=122) | Serum | <3 h | PH ↑ vs. non-PH | Serum c-Fn level ≥3.6 mg/l within 3 h after stroke onset and before tPA therapy predicted PH after tPA therapy with a sensitivity of 100.0%, specificity of 60.0%, and negative predictive value of 100.0%. | Prospective study | Stroke, 2007 |  |
|  | Endostatin | IS (n=29)  CTRL (n=26) | Plasma | <3 h | IS ↑ vs. CTRL | High plasma endostatin level predicted worse long-term functional outcome in IS patients. | Prospective study | Atherosclerosis, 2011 |  |
|  |  | poststroke epilepsy (n=51)  non- poststroke epilepsy (n=844) | Plasma | <6 h | Poststroke epilepsy ↑ vs. non-poststroke epilepsy | A plasma endostatin concentration was higher than 1.203 ng/ml within 6 h after symptom onset could predict the development of epilepsy after a hyperacute stroke event (include IS and HS). | Prospective longitudinal study | Epilepsia, 2020 |  |
|  |  | HS (n=35)  IS (n=154) | Plasma | <4.5 h | IS ↑ vs. HS | N/A | Cohort study | Neurology, 2021 |  |
|  | Caveolin-1 | IS (n=133)  CTRL (n=40) | Serum | <4.5 h | IS ↑ vs. CTRL | A low serum level of caveolin-1 was associated with symptomatic HT after recombinant tPA therapy. | Prospective study | Stroke, 201 |  |
|  | NT-proBNP | HS (n=35)  IS (n=154) | Plasma | <4.5 h | IS ↑ vs. HS | A biomarker panel including RBP4, NT-proBNP and GFAP provided moderate but potentially useful sensitivity rates at 100% specificity for IS diagnosis. | Cohort study | Neurology, 2021 |  |
| Genetic and genomic | miR-223-3p, let-7b-3p | sham (n=12)  Brain ischemia (n=12) | Blood | <4 h | Brain ischemia (miR-223-3p) ↑ vs. sham; Brain ischemia (let-7b-3p) vs. sham ↑ | N/A | Animal research | Acta neurobiologiae experimentalis, 2019 |  |
|  | miRNA-221-3p, miRNA-382-5p | IS (n=78)  CTRL (n=39) | Serum | <6 h | IS vs. CTRL ↑ | N/A | Case-control  study | Journal of Stroke and Cerebrovascular Diseases, 2017 |  |
|  | miR-16 | IS (n=7)  CTRL (n=4) | Plasma | <6 h | IS ↑ vs. CTRL | Plasma miR-16 had an AUC of 0.775 for differentiating IS, with a sensitivity of 69.7% and specificity of 87.0%; and miR-16 AUC, sensitivity and specificity in IS patients reached 0.95, 100.0% and 91.3% in stroke derived from large artery atherosclerosis. | Case-control  study | PLoS One, 2016 |  |
|  | miR-125a-5p, miR-125b-5p, miR-143-3p | IS (n=200)  CTRL (n=100) | Plasma | <6 h | IS ↑ vs. CTRL | The set of plasma miRNAs (miR-125a-5p, miR-125b-5p, miR-143-3p) had an AUC of 0.90 for differentiating IS from healthy control subjects, with a sensitivity of 85.6% and specificity of 76.3%. | Case-control  study | Circ Res, 2017 |  |
|  | circPHKA2 (hsa _circ_0090002), circBBS2 (hsa_circ_0039457) | N/A | Blood | <6 h | N/A | Blood circPHKA2 (hsa _circ_0090002) and circBBS2 (hsa_circ_0039457) were downregulated in IS within 6 h after symptom onset compared to control subjects. | Case-control  study | Front Neurosci, 2020 |  |
|  | tRFs | HS (n=8)  IS (n=9)  SM (n=9) | Plasma | <6 h | IS< SM ↑< HS ↑ | Plasma tRFs had an AUC of 0.986 for differentiating HS from IS and SM. | Cohort study | Int J Mol Sci, 2020 |  |
|  | lncRNA H19 | IS (n=36)  CTRL (n=25) | Plasma | <3 h | IS ↑ vs. CTRL | The plasma H19 had an AUC of 0.910 for differentiating IS from healthy controls, with a sensitivity of 80.6% and specificity of 92.0%. | Case-control  study | Stroke, 2017 |  |
|  | CD40 -1C>T polymorphism (rs1883832) | 222 patients treated with tPA | Blood | <3 h | N/A | CD40-1C>T polymorphism (rs1883832) in peripheral blood was found to be associated with brain vessel reocclusion after fibrinolysis in the early phase (within 3 h) after stroke onset. | Case–case prospective study | Pharmacogenomics, 2010 |  |
|  | Genes | N/A | Blood | <3 h | N/A | A large number of genes showed altered expression in the peripheral blood of humans as early as 3 h after IS onset, which was mainly attributable to neutrophils and was thought to contribute to tissue damage after stroke. | Comparative Study | J Cereb Blood Flow Metab, 2006 |  |
|  |  | N/A | Blood | <3 h  <5 h | N/A | Genes showing altered expression in large-vessel atherosclerotic stroke were associated with platelets and monocytes and are known to be involved in the modulation of hemostasis, whereas those that were altered in cardioembolic stroke were expressed in neutrophils and related to the immune response to infection. | Comparative Study | Journal of Cerebral Blood Flow & Metabolism, 2008 |  |
| Coagulation, thrombosis, metabolism | MPV | 237 patients with IS | Blood | Pre tPA treatment | N/A | Disabling or fatal IS in thrombolytic patients was associated with high blood MPV level before initiation of recombinant tPA treatment. | Retrospective study | Clinical Interventions in Aging, 2019 |  |
|  | Apolipoprotein family (ApoC-I, ApoC-III) | HS (n=15)  IS (n=16) | Plasma | <6 h | IS ↑ vs. HS | N/A | Cohort study | Proteomics, 2004 |  |
|  | BACC  (valine, leucine, isoleucine) | CTRL (n=32)  Mild-IS (n=22)  Severe-IS (n=30) | Plasma | <6 h | Mild-IS vs. CTRL ↑  Severe-IS vs. CTRL ↑ | A similar decrease in BACCs was observed in human plasma within 6±2 h after IS onset, and the degree of reduction was correlated with worse neurologic outcome. | Cohort study | Stroke, 2013 |  |
|  | Proteomic (gelsolin, dihydopyrimidinase-related protein 2 and cystatin A) | good outcome (n=17)  poor outcome (n=43) | Blood | <4.5 h | good outcome vs. poor outcome ↑ | Gelsolin, dihydropyrimidinase-related protein 2 (DPYSL2), and cystatin A in peripheral blood can predict of IS outcome. | Prospective study | Journal of Proteomics, 2013 |  |
|  | Pregnenolone sulfate | IS (n=508)  SM (n=349) | Serum | <3.3 h | N/A | Four metabolites including pregnenolone sulfate outperform CT to differentiate IS and SM. | Prospective study | Ann Neurol, 2020 |  |

Abbreviations: IS: ischemic stroke, OTMT: onset to the measurement time, UCH-L1: ubiquitin C-terminal hydrolase L1, HS: hemorrhagic stroke, SM: stroke mimics, , CTRL: control, AUC: area under the receiver operating characteristic curve, NMDAR: N-methyl-d-aspartate receptor, NfL: Neurofilament light, TIA: transient ischemic attack, GFAP: glial fibrillary acidic protein, S100β: S100 calcium-binding protein B, PD: Parkinson disease, IL-10: Interleukin 10, tPA: tissue plasminogen activator, IL-4R: Interleukin 4 receptor, NLR: Neutrophil to lymphocyte ratio, PLR: platelet to lymphocyte ratio, END: early neurological deterioration, ENI: early neurological improvement, SAP: Serum amyloid protein, SAA: serum amyloid A, HBD-2: Human β-defensin 2, ChT: chitotriosidase, RBP4: Retinol-binding protein 4, FMPPs: Fluorescent molecular peroxidation products, MMP-9: Matrix metalloproteinase-9, PH: parenchymal hematoma, ADMA: Asymmetric dimethylarginine, SDMA: symmetric dimethylarginine, c-Fn: Cellular fibronectin, HT: hemorrhagic transformation, NT-proBNP: N-terminal pro B-type natriuretic peptide, tRFs, tRNA-derived fragments, MPV: Mean platelet volume, ApoC-I: Apolipoprotein CI, ApoC-III: Apolipoprotein CIII, BACC: Branched-chain amino acids, DPYSL2: dihydropyrimidinase-related protein 2, N/A: not applicable due to lack of evidence, ↑: increased level; ↓: decreased level.
